# RE-AIM evaluation of the first 5 years of a citywide produce prescription program

**DOI:** 10.1093/tbm/ibaf057

**Published:** 2025-10-23

**Authors:** Grace Hildebrand, Ronli Levi, Sanjana Marpadga, Ximena Perez-Velazco, Hilary Seligman

**Affiliations:** Division of General Internal Medicine, UCSF, San Francisco, California, United States of America; Joint Medical Program, University of California Berkeley, San Francisco, California, United States of America; School of Medicine, UCSF, San Francisco, California, United States of America; Division of General Internal Medicine, UCSF, San Francisco, California, United States of America; Division of General Internal Medicine, UCSF, San Francisco, California, United States of America; Division of General Internal Medicine, UCSF, San Francisco, California, United States of America; Division of General Internal Medicine, UCSF, San Francisco, California, United States of America

**Keywords:** food insecurity, produce prescription, nutrition, implementation, diet, food is medicine

## Abstract

**Background:**

Food insecurity and poor diet quality increase risk for diet-related chronic disease and contribute to health disparities. Produce prescription programs (PPPs) are designed to promote chronic disease prevention and treatment by lowering barriers to fruit and vegetable (FV) purchases. In collaboration with a network of distribution partners, Vouchers 4 Veggies (V4V) provides eligible participants with vouchers (“prescriptions”) to purchase fresh or frozen FV at participating vendors.

**Purpose:**

To evaluate the implementation and public health impact of the first 5 years of V4V implementation.

**Methods:**

Using the RE-AIM (Reach, Effectiveness, Adoption, Implementation, and Maintenance) framework, we analyzed quality improvement data collected from program participants, distribution partners, and vendors between 2015 and 2020. Participant outcomes included program engagement, FV intake, food security, and program satisfaction; other outcomes included ease of participation and implementation. Data were analyzed using descriptive statistics and *t*-tests.

**Results:**

Between 2015 and 2020, V4V partnered with 135 distribution partners and 29 food vendors to serve 9720 unique participants across San Francisco, representing 10% of the population at high risk for food insecurity. Participants were racially and ethnically diverse. At baseline, 79% reported household food insecurity and 66% reported fair or poor health. At follow-up, food insecurity had decreased by 0.79 points (*P* = .001) on a six-point scale, and FV intake had increased by 0.77 servings/day (*P* = .001). Satisfaction was high among participants, distribution partners, and vendors.

**Conclusions:**

V4V demonstrates the potential for PPPs to improve food security and diet quality across diverse populations for primary and secondary prevention. Sustainable funding and infrastructure are critical for scaling.

## Teaser

Vouchers 4 Veggies is a produce prescription program that provides health care and community organizations with vouchers redeemable for fruit and vegetables (FVs) that are distributed to clients at risk for food insecurity. Evaluation of the program demonstrates that this is a promising strategy to increase FV consumption and decrease food insecurity across a variety of settings and populations.

## Graphical abstract

**Graphical Abstract:**
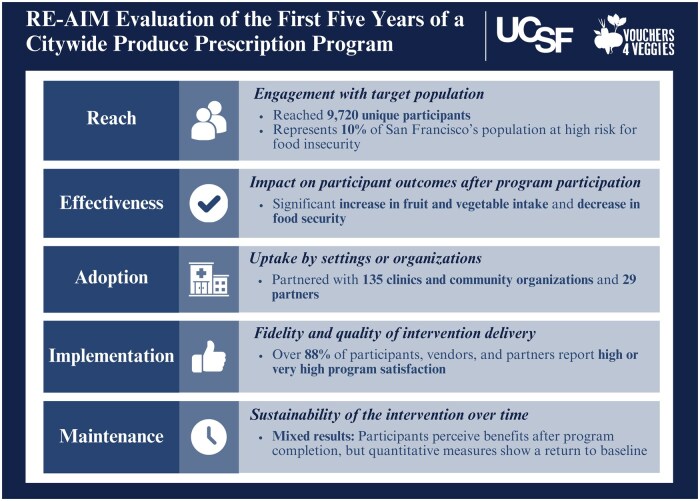

Implications **Practice:** Produce prescription programs (PPPs) are an intervention that may be effective at improving fruit and vegetable consumption and food security that can be offered in conjunction with existing resources in both healthcare and community settings.

**Policy:** The produce prescription model is translatable to settings beyond healthcare and, if expanded, this type of program may fill gaps in the currently inadequate network of federal food assistance and charitable food assistance through community-led outreach.

**Research:** Investigators should further research the implementation of PPPs across different settings and populations to establish best practices.

## Introduction

Only 10%–12% of adults in the United States meet the Dietary Guidelines for Americans recommendations for fruit and vegetable (FV) intake [1]. Insufficient consumption of these nutrient-dense foods is linked to a higher risk of chronic diseases, including cardiovascular disease, type 2 diabetes, and certain cancers. These chronic diseases contribute to the nation’s growing burden of diet-related mortality; poor diet is a leading cause of death in the US and contributes to over 50 billion dollars in costs related to cardiometabolic disease [2]. Although most Americans have poor dietary intake, low-income individuals face additional barriers to healthy dietary intake compared with higher-income individuals, including higher food costs, and limited access to affordable healthy options [3]. These structural and economic barriers can negatively impact dietary intake, exacerbating health disparities and increasing the risk of poor health outcomes in underserved communities [3].

There has been increasing national support for “food is medicine” (FIM) initiatives, such as produce prescription programs (PPPs), which aim to simultaneously address food and nutrition insecurity by reducing barriers to accessing healthy produce [4]. Traditionally, these interventions are carried out in close collaboration with healthcare providers. PPPs, usually provided as a voucher, electronic debit card, or produce box, are “prescribed” to individuals at risk for or experiencing a diet-related health condition or food insecurity [5]. By increasing the affordability and accessibility of FVs, PPPs can help reduce economic barriers to healthy eating and improve food security and diet quality [6]. Sustained participation may lead to both short- and long-term health benefits (including better weight management, glycemic control among people with diabetes, and cardiovascular health), underscoring the role PPPs can play in promoting health equity [7].

Effective implementation is critical for translating evidence-based interventions into real-world settings, but there is limited research on PPPs that applies structured implementation science frameworks or examines implementation in depth. This is particularly important for PPPs because of the complexity of implementation, which requires engagement and capacity-building across multiple sectors (health care, food vendors, and community-based organizations). Barriers identified in the literature for program implementers include data sharing and privacy concerns in partnering with food vendors, limited integration into existing clinical workflows, and limited staff capacity or resources to support implementation [8]. Common facilitators for implementation include adequate staffing and flexible workflows [8, 9]. For participants, commonly reported barriers include transportation difficulties, lack of program usability, lack of choice, cultural barriers, stigma, and inconvenient retail locations [6, 10, 11]. Facilitators include robust support services and communication, longer program durations, and a greater number of shopping locations [6, 10]. However, most PPP studies have been conducted in the healthcare setting, relying primarily on stakeholder interviews to describe barriers and facilitators. Few studies assess program drivers and protocols or examine multiple implementation domains, including outcomes, simultaneously [8, 9, 12]. To better understand the real-world impact of PPPs, research is needed to evaluate their feasibility, implementation, sustainability, and scalability across diverse settings.

Vouchers for Veggies (V4V) is a PPP established in San Francisco in 2015. Since its inception, V4V has systematically collected data to assess program effectiveness and implement rapid quality improvement initiatives. Although research has been conducted on the program’s impact in specific populations, there has not been a formal study of the program’s implementation or its public health impact across multiple settings and populations [13, 14].

RE-AIM (Reach, Effectiveness, Adoption, Implementation, and Maintenance) is an implementation science framework that can guide intervention planning and structure reporting of an intervention’s impact [15]. RE-AIM has been widely used to evaluate behavioral interventions. It allows for systematic assessment of an intervention’s effectiveness, implementation, sustainability, and scalability across diverse populations and contexts [16]. In this study, we apply RE-AIM as an evaluation framework to assess the effects of V4V across multiple domains and settings, including individual and organizational settings. Given the growing interest in scaling PPPs, RE-AIM offers a structured approach to assess V4V’s real-world impact and identify factors contributing to its success.

## Methods

V4V provides benefits, in the form of paper or electronic vouchers, to eligible individuals to purchase fresh or frozen FVs at a network of retailer locations that includes large grocery chains, farmer’s markets, and corner stores across San Francisco. Vendors are chosen based on their proximity to where participants live, work, or receive services and their interest in participation. V4V vendors must meet explicit criteria for affordability, variety, and quality of produce before being accepted as redemption sites.

Unlike most PPPs that operate entirely in partnership with healthcare, V4V partners with community-based organizations and clinics (referred to as “distribution partners”) to enroll eligible participants and distribute vouchers. Participant eligibility criteria are broad and include people with and without a chronic disease diagnosis; they are determined based on funding priorities and in collaboration with distribution partners. Eligibility screening approaches vary and are at the discretion of the distribution partners. This approach allows distribution partners to identify and serve those most in need. In some settings, such as health clinics, a formal approach is used, where eligibility is determined based on a participant’s diagnosis, such as diabetes or pre-diabetes. In other settings, such as low-income supportive housing sites, distribution partners may use a categorical eligibility approach, where participants are automatically deemed eligible because they meet certain income thresholds by being residents of that site. V4V also partners with all Special Supplemental Nutrition Program for Women, Infants, and Children (WIC) clinics across San Francisco to enroll pregnant WIC participants.

This study uses the RE-AIM framework to systematically examine program data collected between January 2015 and June 2020. We determine the impact of V4V across the framework’s five dimensions: reach, effectiveness, adoption, implementation, and maintenance [15]. While our internal processes assessed some aspects of each of the five dimensions, this is a pragmatic evaluation based on data collected for quality improvement efforts, and thus, the robustness of measures in each dimension varies.

Reach assesses the extent to which the program engages its intended target population, including demographic characteristics and potential disparities in access. Effectiveness evaluates the program’s impact on health-related outcomes, such as dietary behaviors and food security. Adoption examines how distribution partners and food vendors integrate the program into their services, identifying factors that influence participation at the institutional level. Implementation considers how consistently and effectively the program is delivered, including fidelity to its intended design, adaptations made, and barriers to execution. Maintenance analyzes the long-term sustainability of the program at both the participant and organizational levels, determining whether access to the program persists over time and whether it remains integrated into community and healthcare settings.

This evaluation uses data only from San Francisco, although V4V has been replicated in other locations across California and the US. The study was approved by the institutional review board at University of California, San Francisco.

### Measures and data sources

Participant, distribution partner, and vendor data previously collected as part of ongoing programmatic activities and quality improvement efforts were organized into each dimension of the RE-AIM framework. Below, we describe each primary data source in more detail. Definitions of measures and data sources for each dimension are presented in Table 1. A detailed description of each survey item can be found in the Appendix.

**Table 1:** RE-AIM measures and data sources

Dimension	Re-AIM definition	Level	Measures	Data sources
Reach	The absolute number, proportion, and representativeness of individuals who are willing to participate in program	Participant	Absolute number of unique program enrollments Proportion of unique participants (numerator) reached by the program out of the total eligible San Francisco population at or below 200% Federal Poverty Line (denominator) Participant demographics (race, ethnicity, income, household size, gender, and age) Representativeness of program participants reached compared to San Francisco population at or below 200% Federal Poverty Line	Enrollment forms (numerator) U.S. Census Bureau American Community Survey 5-year estimate: Poverty status in the past 12 months of families (denominator) California Health Interview Survey (2015–2020): demographic characteristics of individuals at or below 200% FPL (representativeness)
Effectiveness	The impact of program on food security, dietary intake, and health	Participant	Household food security status Fruit and vegetable consumption Self-reported health status (“in general, would you say your health is: excellent, very good, good, fair, or poor?”) Self-reported changes in eating habits after program participation (follow-up survey only): variety of fruits and vegetables consumed, confidence in ability to make healthy food choices on a budget, knowledge of fruits and vegetables, consumption of unhealthy foods	Baseline and follow-up surveys
		Setting	Impact on vendor sales (e.g. changes in store and produce sales; changes in re-stocking of fruits and vegetables due to program participation) and customer base (e.g. increases in new or repeat customers) Distribution partner assessment of program utility (“how helpful is V4V as a resource for your clients? Very helpful, helpful, somewhat helpful, not helpful”)	Vendor and distribution partner surveys
Adoption	Number of vendors and distribution partners willing to initiate and deliver the program	Setting	Total number and types of distribution partners Total number and types of participating vendors	Organizational records
Implementation	Participant experience using the program, and vendor and distribution partner experience implementing the program according to protocol	Participant	Participant satisfaction (“please rate your level of satisfaction with V4V: very high, high, average, low, very low”) Voucher redemption: proportion of voucher dollars redeemed (numerator) over voucher dollars distributed (denominator)	Follow-up surveys Voucher redemption data
		Setting	Vendor and distribution partner satisfaction Vendor net promoter score Distribution partner ease of enrollment and voucher distribution Vendor ease of voucher acceptance and processing	Vendor and distribution partner surveys
Maintenance	The extent to which outcomes are maintained 6 or more months after program completion, and the extent to which V4V becomes institutionalized within vendors, partners, and city-wide	Participant	Extent to which effectiveness (fruit and vegetable intake, food security, and self-reported health) is sustained after program completion	Participant maintenance surveys
		Setting	Length of time vendors and distribution partners work with V4V	Vendor and distribution partner surveys Organizational records

#### Participant data

We collected sociodemographic data for all participants at program enrollment. We administered complete baseline and follow-up surveys to sub-samples of participants at enrollment and after 4–6 months, measuring outcomes including FV intake (measured using a validated seven-item screener), food security [measured using the United States Department of Agriculture’s (USDA) six-item Food Security Survey Module], participant satisfaction, and self-reported health [17, 18]. Surveys were available in three languages (English, Spanish, and Chinese) and either self-administered or completed with the help of distribution partner staff, generally due to translation needs, vision impairments, or limited literacy. Although enrollment data were collected through June 2020, we ended survey data collection in March 2020 due to the onset of the COVID-19 pandemic.

Survey data includes unique participants and participants who re-enrolled during the study period, provided their subsequent enrollment occurred at least 6 months after their previous one. Between 2015 and 2020, we collected 1388 matched baseline and follow-up food insecurity screeners, 1380 matched FV screeners, and 1528 matched self-reported health surveys from 1613 unique participants. We also collected 2252 surveys on self-reported items only asked on follow-up, which included self-reported changes in eating habits after program participation (variety of FVs consumed, confidence in making healthy food choices on a budget, knowledge of FVs, and consumption of unhealthy foods). Survey data for this study excludes participants enrolled through WIC, as those results have been reported elsewhere [13].

In 2017, we administered maintenance surveys to 128 former participants who met language and eligibility criteria (see Appendix), assessing the sustainability of healthy eating, food security, and health 6–12 months after program participation ended.

To determine voucher redemption rates, we used data on voucher dollars redeemed (numerator) and voucher dollars distributed (denominator).

#### Vendor data

We report data on the number and types of food vendors where vouchers could be redeemed during the study period. In addition, we collected cross-sectional surveys from participating vendors in 2017 and 2019, with 46 responses from 29 unique vendors. These surveys included questions assessing ease of processes, consumer benefits, and store impact.

#### Distribution partner data

We used organizational records to determine the number and type of distribution partners V4V collaborated with during the study period. Cross-sectional surveys were collected from distribution partners in 2016 and 2019 and included questions measuring ease of program implementation, benefits to clients, and impact on their organization. For the analysis, we combined surveys from both years of data collection, totaling 67 survey responses. Since data were collected anonymously, it may include duplicate responses.

### Data analysis

We used descriptive statistics to assess program reach and self-reported participant outcomes, including food security, changes in eating habits, and health. Due to the broad eligibility criteria, we used ≤200% of the federal poverty level (FPL) in San Francisco as the denominator to calculate the representativeness of program participants. While we did not have specific income thresholds for program participation, many participants were enrolled in programs designed to support individuals with low incomes.

Program reach was calculated by dividing the program’s unique household enrollments over the study period by the number of San Francisco households estimated to have incomes ≤200% FPL. For total households ≤200%, we used the US Census Bureau’s 2019 ACS 5-year estimate of poverty status in the past 12 months of families, which represents a weighted average of survey responses collected over a 5-year period (a pooled period estimate, not a cumulative total).

The USDA’s six-item Food Security Survey Module was scored by summing affirmative responses to determine a raw score between 0 and 6. We replaced the raw score with the USDA’s Rasch-computed scale score, because a continuous score offers greater power to detect the severity of food insecurity when compared to a categorical or dichotomous measure of food security. However, we also used the more common scoring algorithms to create a dichotomous variable of food security that considers two or more affirmative responses to represent food insecurity, and a categorical variable of high (no affirmative responses), marginal (1 affirmative response), low (2–4 affirmative responses), or very low (5–6 affirmative responses) food security.

We calculated the average daily servings of FV consumption per participant using the standard algorithm associated with the dietary intake screener [17]. We excluded surveys from our analysis that had more than one missing response or were considered outliers. Notably, in the initial 3 years of survey completion, a question related to tomato consumption was inadvertently omitted from all surveys. As a result, baseline and follow-up surveys (*n* = 438) were missing one of the seven items. We assigned missing tomato values based on the average baseline and follow-up tomato consumption of participants who received the complete seven-item screener to correct this systematic omission. We also conducted a sensitivity analysis without the imputed tomato question.

We report descriptive statistics on program satisfaction (among participants, vendors, and distribution partners), as well as on ease of program implementation, benefits to clients, and organizational impact (among vendors and distribution partners). Net promoter score (NPS) is a metric commonly used to measure “customer loyalty.” We calculated NPS based on vendor responses to the question “How likely are you to recommend Vouchers 4 Veggies to a friend or colleague?” NPS was scored on a zero to 10 scale using the standard categories of promoters (9–10), passives (7–8), and detractors (0–6). To calculate NPS, we subtracted the percentage of detractors from the percentage of promoters, which can result in a score ranging from −100 to 100, with a higher score being better [19].

Paired-sample *t*-tests were conducted to test the statistical significance of changes in continuous measures of FV intake and food security status between baseline and 6 months and again 6−12 months after program completion among a subsample of program participants with available data. Wilcoxon signed-rank tests were used to test the statistical significance of categorical variables. McNemar’s test was used to test for statistical significance for paired binary variables. All *P*-values are reported at the conventional significance level of <.05. Analyses were performed using Stata software (version 16 StataCorp, College Station, TX, United States) and Microsoft Excel.

## Results

### Reach

During the study period, V4V provided FV vouchers to 9720 unique participants. Among those, 1659 participants participated in the program more than once. Participants were considered duplicates if they re-enrolled (which occurs at the discretion of the distribution partner) or received vouchers from another organization after their initial enrollment ended. Including duplicate participants, there were a total of 11 379 participants during the study period. On average, participants enrolled 1.2 times (standard deviation, SD = 0.53).

In 2015, we estimate V4V reached 0.86% of San Francisco households with incomes ≤200% of the FPL. In 2019, reach increased to 3.76%. Overall, we estimate that V4V served approximately 10% of the estimated 92 186 households ≤200% FPL in San Francisco at some point between 2015 and 2020.

We present baseline participant characteristics in Table 2. The sample was racially and ethnically diverse, with a majority living in households of one to two people (73%) and households experiencing food insecurity (79%). Over a quarter (27%) of participants were pregnant and enrolled in WIC.

**Table 2:** Participant characteristics and characteristics of eligible population at baseline

Variable	Participants, *n* (%)^a^	San Francisco population below 200% FPL, *n* (%)^b^
Race and ethnicity, *n* = 9720		
Latino or Hispanic	1932 (23.5)	62 000 (25.6)
White or Caucasian	1140 (13.9)	41 000 (19.5)
Black or African American	1852 (22.5)	21 000 (11.3)
Asian American or Pacific Islander	2960 (36.0)	82 000 (39.9)
Another race or ethnicity	349 (4.2)	6000 (2.9)
Monthly income, *n* = 6294		N/A
None	366 (7.0)	
Less than $500	632 (12.1)	
$501–$1000	2535 (48.5)	
$1001–$2000	1375 (26.3)	
$2000 or more	318 (6.1)	
Household size, *n* = 8296		
1–2 people	5255 (73.4)	75 000 (37.3)
3 or more people	1907 (26.6)	147 800 (62.7)
Gender, *n* = 6294		N/A
Male	2237 (42.8)	
Female	2908 (55.7)	
Another gender	80 (1.5)	
Age, *n* = 9720		
Under 18	8 (<1)	43 000 (19.6)
18–39	3061 (33.7)	58 000 (26.8)
40–64	3177 (35.0)	56 000 (26.5)
65+	2829 (31.2)	45 000 (23.4)
Eligibility status, *n* = 9720		
Pregnant person	2647 (27.2)	N/A
Food security, *n* = 1344		
Food insecure	1056 (78.6)	78 000 (44.9)
Food secure	288 (21.43)	63 000 (55.1)
Health status, *n* = 1554		N/A
Poor	256 (17%)	
Fair	773 (50%)	
Good	387 (25%)	
Very good	99 (6%)	
Excellent	31 (2%)	

The percentages reported are based only on valid responses, excluding blank or incomplete submissions. However, the reported sample size (*n*) includes all responses, including those that were left blank.

a Participant *N* varies due to differences in data source.

b Data are estimates derived from the California Health Interview Survey. All categories besides food insecurity are from 2015 to 2020 for San Francisco. Food insecurity data are only available from 2017 and 2018 and combines data from San Francisco and Alameda counties.

### Effectiveness

There was a statistically significant mean improvement of 0.79 points in household food security on a six-point scale from baseline to follow-up [*n* = 1139, 95% confidence interval (CI): 0.60–0.98, *P* < .001). At baseline, 79% reported food insecurity, decreasing to 70% at follow-up (*n* = 1344, *P* < .001). Additionally, 35% moved up at least one food security category in the four-level categorical variable (*n* = 1139, *P* < .001). Although 44% of V4V participants experienced very low food security at baseline, only 32% reported very low food security at follow-up.

Mean FV consumption increased by 0.77 servings per day from baseline to follow-up (*n* = 1261, 95% CI: 0.65–0.89, *P* < .001), from an average of 2.62 servings to 3.39 servings. In a sensitivity analysis, results were similar without imputing the tomato question; mean consumption increased by 0.71 servings per day (*n* = 1261, 95% CI: 0.61–0.82, *P* < .001).

At program completion, most participants (*n* = 2252) reported healthy changes in eating habits: 97% increased their variety of FVs, 98% felt more confident in making healthy budget choices, 95% improved their knowledge of FVs, and 89% ate fewer unhealthy foods.

Participants (*n* = 1554) also reported improved general health, with 39% of respondents rating their health at least one category higher at follow-up (*P* < .001). The proportion of participants reporting good to excellent health increased from 34% at baseline to 49% at follow-up. Additionally, 93% of 2094 participants agreed or strongly agreed that their health improved after participating in V4V.

Almost all (97%) distribution partners surveyed viewed V4V as a helpful or very helpful resource for their clients. Vendors (*n* = 46 surveys) perceived V4V to positively impact their customer base, produce sales, and overall revenue: 73% reported increased income, 89% reported higher FV sales, 75% reported more customers, 85% noted an increase in new customers, and 89% reported having more repeat customers. Furthermore, 43% of vendors reported that they began stocking new produce, and 39% displayed more FVs as a direct result of V4V. In 2019, 58% of vendors surveyed (*n* = 26) reported selling higher-quality produce.

### Adoption

Between 2015 and 2020, V4V worked with 135 unique distribution partners in San Francisco. Distribution partners included affordable housing sites (69%), public health and community clinics (14%), other community-based organizations (12%), and WIC clinics (5%). By the end of the study period, V4V had expanded its vendor network to 29 vendors, comprised of small and mid-sized markets (*n* = 23), chain grocery stores (*n* = 4), and farmers markets (*n* = 2) across 15 San Francisco neighborhoods.

### Implementation

Participants (*n* = 2252) expressed high satisfaction with the program (88%) and found the vouchers easy to redeem (95%). Approximately two-thirds of participants found the voucher amounts appropriate, while 32% considered the amount too low. Voucher redemption rates ranged from 74% to 76%.

Almost all distribution partners found it easy to identify (94%) and enroll (95%) participants. Among distribution partners surveyed in 2019 (*n* = 34), 97% expressed high or very high satisfaction with V4V, and 97% found it easy to distribute vouchers. These questions were not previously asked in 2016.

Vendors were generally satisfied with V4V, with 89% of respondents reporting that they were extremely or very satisfied and an overall NPS of 85. Further, 94% of vendors found vouchers easy to accept at their stores, and 98% found the program easy to participate in. While the majority (95%) of vendors found program staff to be helpful and responsive to their needs, only 73% reported that they were reimbursed quickly for vouchers accepted in their stores.

### Maintenance

Among a subset of participants (*n* = 128) surveyed 6–12 months after program completion, 66% reported that their post-­program FV consumption increased or remained consistent with levels during program enrollment using a single-item assessment of intake. However, FV screener results showed intake returned to near baseline levels after program completion. Similarly, food security and self-perceived health results returned to baseline levels 6–12 months after program completion.

V4V generally determines which distribution partners can continue the program over subsequent years because participation is determined by available funding. As of 2019, funding allowed 50% of surveyed distribution partners to report voucher distribution for over 1 year. This suggests a degree of program maintenance, though future evaluations with more robust longitudinal data are needed to better assess partner retention over time.

Vendors reported an ongoing impact from V4V on their customer base, with 85% noting that customers continued shopping at their store even after no longer participating in the program. Only one vendor decided to stop working with V4V in the 5 years of program operation.

## Discussion

In an implementation evaluation of the first 5 years of a PPP based in San Francisco, CA, we found that the program achieved broad reach and statistically significant increases in household food security, FV intake, and self-reported health. Though effects on FV consumption were modest, research suggests that increases in FV consumption can have an effect on health even at small doses, particularly when baseline consumption is low [20]. Additionally, we observed improvements in self-reported health status, aligning with findings from other produce prescription evaluations [21]. The single-­item self-reported health status question can serve as a proxy for health-related quality of life and correlates with morbidity and mortality risk [22].

This study demonstrates a PPPs effectiveness in clinical settings and community-based environments such as supportive housing units, senior centers, and family resource centers. Although most existing studies of PPPs are conducted among clinical populations enrolled in clinical settings, our findings are consistent with research conducted in these more narrow populations [6, 7, 23]. For example, a pooled analysis of nine PPPs across 12 US states found FV intake increased by 0.85 cups per day (95% CI: 0.68–1.02), similar to the magnitude of our findings [24]. However, because our analysis was pooled across diverse populations and settings, our ability to directly compare results with more narrowly focused clinical studies is limited. A recent scoping review of community-based incentive programs also found that providing produce prescriptions in various community settings outside of healthcare can improve FV intake, food security, and specific health outcomes [25]. Future research should further investigate program impacts within specific subgroups and settings to enhance comparability and guide more tailored interventions.

While our data does not allow us to assess fidelity, we were able to determine that distribution partners and vendors were highly satisfied with the intervention. We calculated an NPS of 85 among participating vendors, indicating a high level of loyalty and satisfaction. Both vendors and distribution partners found the program easy to implement, and changes to the program model have since addressed many of the challenges identified over time. For instance, the program now offers a debit card option in addition to paper vouchers, streamlining grocery check-out. The ease of implementation and high satisfaction and redemption rates suggest strong partner support for scaling this program.

Maintenance of program results is mixed. While participants perceived that they maintained FV consumption after program participation ended, assessment of FV intake and food security using validated scales revealed a return to baseline levels. The extent to which the effects of PPPs are maintained on an individual level after program completion is a well-documented gap in the literature [26]. Qualitative research has found that participants struggle to maintain the same levels of FV consumption following program completion [27].

Program sustainability is also a key component of program maintenance. An important indicator of sustainability is the extent to which the program is institutionalized within implementing partners [28]. Both distribution partners and vendors have continued to work with V4V past this evaluation’s end date, indicating high program buy-in and maintenance of infrastructure for voucher distribution and redemption. Since the formal evaluation ended in 2020, V4V has continued to expand its reach. As of 2024, V4V partners with 55 small, local vendors and grocery chains and 247 distribution partners across San Francisco. This sustained growth suggests a high potential for sustainability.

Funding remains a primary obstacle for PPPs. Many programs rely on short-term grants, hindering their ability to expand beyond the pilot phase and maintain consistent operations. Likewise, V4V faces sustainability challenges primarily due to funding constraints, despite high participant demand and high distribution partner and vendor satisfaction. V4V has been able to braid funding from governmental and nongovernmental sources to increase reach and serve a variety of populations. To make the most of limited funding, V4V typically works with numerous distribution partners for a single program cycle rather than working with fewer distribution partners for multiple program cycles. However, the latter can limit program continuity, potentially leaving resource gaps post-participation.

### Strengths, limitations, and future directions

Our evaluation was limited by its use of programmatic, quality improvement data that prioritizes improving service delivery and addressing immediate needs rather than research. As such, we have missing demographic data and loss to follow-up. This missingness limits our conclusions from the demographic data and may bias our effectiveness results. Further, our outcome measures were self-reported variables that may introduce social desirability and recall biases. The measures were evaluated using a pre-/post-design without a control or comparison group, which precludes causal conclusions about effectiveness. This study was also conducted in a single city strongly committed to supporting food and nutrition security, leveraging dedicated municipal funding. These contextual factors may limit its generalizability to other cities across the United States. Because V4V is implemented in the community in addition to clinical settings and does not always require a chronic disease diagnosis, our participants were likely healthier than most studies of PPPs. Thus, our evaluation should be understood as focusing more on primary prevention, compared with studies of more clinical populations that focus more on secondary prevention. Finally, while RE-AIM provides a structured approach to evaluation, successful translation into practice requires that an implementation science framework be applied during the planning process.

These limitations arise from trade-offs that also speak to the strengths of our study. First, despite loss to follow-up, 5 years of records provided us with a large sample. The burden of data collection and evaluation has been repeatedly described in the literature as an implementation challenge of PPPs, and the ability to utilize real-world data for research balances prioritizing the needs of implementing partners while also rigorously evaluating the program. Using implementation science frameworks such as RE-AIM, which are designed to be applied to flexible data sources, supports the strength of the conclusions. Finally, we evaluate a program scaled across an entire city and encompassing diverse community settings and populations.

Future research should continue to assess the effectiveness of PPPs across diverse populations, identify barriers and facilitators to implementation in various settings (including community and healthcare environments), and examine the maintenance of program effects over time. Additionally, studies should explore how different program models and components influence outcomes to optimize impact and scalability.

## Conclusion

PPPs like V4V can have broad reach and demonstrate some evidence of effectiveness. They are also highly scalable if sustainable, multi-year funding is secured. V4V’s growth is attributable, among other factors, to a corresponding increase in funding. In recent years, there has been policy momentum to develop sustainable funding for PPPs, such as those funded by the USDA’s Gus Schumacher Nutrition Incentive Program and through Medicaid reimbursement pathways [21, 29]. Most policy proposals focus on funding and implementation in healthcare settings, but this study suggests the model is translatable to settings beyond healthcare. If expanded, PPPs may fill gaps in the currently inadequate network of federal and charitable nutrition support. As funding grows for PPPs, it is critical that other studies examine the implementation of these programs across different settings and populations in order to establish best practices. This evaluation can inform policymakers, public health officials, and community leaders in making strategic decisions about funding, adapting, and scaling PPPs to maximize their public health impact.

## Funding Sources

Funding support for Vouchers 4 Veggies during the time period described was provided by the Hellman Foundation and the San Francisco Department of Public Health, as well as numerous others small and individual donors. The research was supported by the Diabetes Research for Equity through Advanced Multilevel Science (DREAMS) CDTR (P30DK092924). The funders had no role in the design and conduct of the study; collection, management, analysis, and interpretation of the data; preparation, review, or approval of the manuscript; or decision to submit the manuscript for publication.

## Welfare of Animals

Not applicable. This article does not contain any studies with animals performed by any of the authors.

## Transparency Statements

### Study Registration

This study was not registered as it utilized existing quality improvement program records for analysis.

### Analytic Plan Registration

An analytic plan was not preregistered for this study, as it was based on retrospective analysis of existing data.

### Availability of Data

The data used in this study are derived from existing quality improvement program records and can be made available to qualified researchers upon request.

### Availability of Analytic Code

The analytic code used for this study is available upon request to qualified researchers.

### Availability of Materials

Materials relevant to the study, including datasets and analytic code, can be made available to qualified researchers upon request, subject to appropriate privacy and compliance standards.

## Appendix 1 Measuring the use of the RE-AIM model dimension items checklist (adapted from http://cancercontrol.cancer.gov/is/)

**Table:**

Dimensions/items	Included? (Yes, no, yes—Inappropriate use, N/A)
**Reach**	
Exclusion criteria (% excluded or characteristics)	**No.** *The program has no explicit exclusion criteria, as inclusion/exclusion depends on the missions and populations of distribution partners. Example inclusion/exclusion criteria of partner sites include those serving individuals who are pregnant, low-income, food insecure, and/or are experiencing chronic disease (such as diabetes, prediabetes, or hypertension).*
Percent of individuals who participate, based on valid denominator	**Yes.** *Percent of individuals reached compared to priority population of individuals at risk for food security, approximated by federal poverty level at or below 200% federal poverty limit (FPL).*
Characteristics of participants compared to nonparticipants or to target population	**Yes.** *Reported in Table 2*.
Use of qualitative methods to understand reach and/or recruitment.	**No.** *We collected qualitative data, but do not report it in this manuscript.*
**Effectiveness**	
Measure of primary outcome with or without comparison to a public health goal	**Yes**. *We report primary outcomes of food insecurity and fruit and vegetable consumption.*
Measure of broader outcomes (e.g. other outcomes, measure of QoL or potential negative outcome) or use of multiple criteria	**Yes.** *We report changes in self-reported health status and perceived changes in eating behaviors for participants. For distribution partners, we report the perceived value of the benefit. For vendors, we report programmatic impact on their store.*
Measurement of robustness across subgroups	**No.**
Measurement of short-term attrition (%) and differential rates by patient characteristic or treatment condition.	**Yes, partially.** *Attrition is approximated by percentage of distributed vouchers redeemed at stores. Data does not allow for presentation of attrition rates by sub-group.*
Use of qualitative methods/data to understand outcomes.	**No.** *We collected qualitative data but do not report it in this manuscript.*
**Adoption (setting level)**	
Setting exclusions (% or reasons)	**Yes, partially.** *The program has no explicit requirement for distribution partners, so long as they serve eligible participants. Vendors are chosen based on their proximity to where participants live, work, or receive services and their interest in participation. V4V vendors must meet explicit criteria for affordability, variety, and quality of produce before being accepted as redemption sites.*
Percent of settings approached that participate (valid denominator)	**No.** *There is greater demand for the program than their funding capacity. Distribution partner participation is based on funding requirements and availability.*
Characteristics of settings participating (both comparison and intervention) compared to either: nonparticipants or some relevant resource data	**Yes.** *Characteristics of vendors and distribution partners are described. There is no external data source that allows for a comparison to nonparticipating vendors and distribution sites.*
Use of qualitative methods to understand adoption at setting level	**No.** *We collected qualitative data, but do not report it in this manuscript.*
**Adoption—Staff level**	
Staff level staff exclusions (% or reasons)	**N/A**
Percent of staff invited that participate	**N/A**
Characteristics of staff participants versus nonparticipating staff or typical staff	**N/A**
Use of qualitative methods to understand staff participation implementation	**No.** *We collected qualitative data, but do not report it in this manuscript.*
**Implementation**	
Percent of perfect delivery or calls completed	**No.** *We did not formally evaluate this.*
Adaptations made to intervention during study	**No.** *We did not formally evaluate this.*
Cost of intervention (time or money)	**No.**
Consistency of implementation across staff/time/settings/subgroups	*We did not formally evaluate this.*
Use of qualitative methods to understand implementation	**No.** *We collected qualitative data, but do not report it in this manuscript.*
**Maintenance—Individual level**	
Measure of primary outcome (with or w/o comparison to a public health goal) at ≥ 6-month follow-up after final intervention contact	**Yes**
Measure of broader outcomes or use of multiple criteria at follow-up (e.g. measure of QoL or potential negative outcome)	**No.** *We collected additional outcomes in the maintenance survey, but they are not reported in this manuscript.*
Robustness data—something about subgroup effects over the long term	**No.**
Measure of long-term attrition (%) and differential rates by patient characteristics or treatment condition	**No.**
**Maintenance—Setting level**	
If program is still ongoing at ≥6 month post-study funding	**N/A**

## Appendix 2 Survey questions

**Table:**

Survey type	Outcome	Question	Response options	Distribution frequency	Domain
Participant	Fruit and vegetable intake	In the last 4 weeks how often did you drink fruit juice (like orange, apple, or grape)? Include fresh, frozen or canned juice but not soda or other drinks.	Less than once per week About 1 time per week 2–3 times per week 4–6 times per week Once per day 2 or more times per day	Baseline, follow-up, and maintenance surveys	Effectiveness
Participant	Fruit and vegetable intake	In the last 4 weeks how often did you eat any fresh fruit, canned fruit, or fruit in smoothies? Don’t count juice.	Less than once per week About 1 time per week 2–3 times per week 4–6 times per week Once per day 2 or more times per day	Baseline, follow-up, and maintenance surveys	Effectiveness
Participant	Fruit and vegetable intake	In the last 4 weeks how often did you eat green salad (like lettuce or spinach salad)?	Less than once per week About 1 time per week 2–3 times per week 4–6 times per week Once per day 2 or more times per day	Baseline, follow-up, and maintenance surveys	Effectiveness
Participant	Fruit and vegetable intake	In the last 4 weeks how often did you eat tomatoes or salsa fresca?* ***Question excluded during first 3 years of survey administration	Less than once per week About 1 time per week 2–3 times per week 4–6 times per week Once per day 2 or more times per day	Baseline, follow-up, and maintenance surveys	Effectiveness
Participant	Fruit and vegetable intake	In the last 4 weeks how often did you eat vegetable soup or stew with vegetables?	Less than once per week About 1 time per week 2–3 times per week 4–6 times per week Once per day 2 or more times per day	Baseline, follow-up, and maintenance surveys	Effectiveness
Participant	Fruit and vegetable intake	In the last 4 weeks how often did you eat potatoes of any kind, including baked, mashed, or French fried?	Less than once per week About 1 time per week 2–3 times per week 4–6 times per week Once per day 2 or more times per day	Baseline, follow-up, and maintenance surveys	Effectiveness
Participant	Fruit and vegetable intake	In the last 4 weeks how often did you eat any other vegetables, including green beans, peas, corn, and broccoli?	Less than once per week About 1 time per week 2–3 times per week 4–6 times per week Once per day 2 or more times per day	Baseline, follow-up, and maintenance surveys	Effectiveness
Participant	Food security	“The food that we bought just didn’t last, and we didn’t have money to get more.” Was that often, sometimes, or never true for you/your household in the last 30 days?	Often true Sometimes true Never true Don’t know	Baseline, follow-up, and maintenance surveys	Effectiveness
Participant	Food security	“We couldn’t afford to eat balanced meals.” Was that often, sometimes, or never true for you/your household in the last 30 days?	Often true Sometimes true Never true Don’t know	Baseline, follow-up, and maintenance surveys	Effectiveness
Participant	Food security	In the last 30 days, did you ever cut the size of your meals or skip meals because there wasn’t enough money for food? If yes, how many days did this happen?	Yes, 3 days or more (in the last 30 days) Yes, only 1–2 days (in the last 30 days) No (in the last 30 days) Don’t know	Baseline, follow-up, and maintenance surveys	Effectiveness
Participant	Food security	In the last 30 days, did you ever eat less than you felt you should because there wasn’t enough money for food?	Yes No Don’t know	Baseline, follow-up, and maintenance surveys	Effectiveness
Participant	Food security	In the last 30 days, were you ever hungry but didn’t eat because there wasn’t enough money for food?	Yes No Don’t know	Baseline, follow-up, and maintenance surveys	Effectiveness
Participant	Self-reported health status	In general, would you say your health is:	Poor Fair Good Very good Excellent Don’t know	Baseline, follow-up, and maintenance surveys	Effectiveness
Participant	Self-reported changes in eating habits after program participation	Because of the Vouchers 4 Veggies program, I eat more kinds of fruits and vegetables	Strongly agree Agree Somewhat disagree Disagree	Follow-up survey	Effectiveness
Participant	Self-reported changes in eating habits after program participation	Because of the vouchers 4 program, I am more confident in my ability to make healthy food choices on a budget	Strongly agree Agree Somewhat disagree Disagree	Follow-up survey	Effectiveness
Participant	Self-reported changes in eating habits after program participation	Because of the Vouchers 4 Veggies program, my knowledge of the importance of fruits and vegetables in my diet has improved	Strongly agree Agree Somewhat disagree Disagree	Follow-up survey	Effectiveness
Participant	Self-reported changes in eating habits after program participation	Because of the Vouchers 4 Veggies program, I am eating less unhealthy food (like chips, cookies, fast food, etc.).	Strongly agree Agree Somewhat disagree Disagree	Follow-up survey	Effectiveness
Vendor	Satisfaction	Overall, how satisfied are you with Vouchers 4 Veggies?	Extremely satisfied Very satisfied Moderately satisfied Slightly satisfied Not all satisfied	Cross-sectional, 2017 and 2019	Implementation
Vendor	Produce sales	Because of the program, my store sells more fruits and vegetables	Yes No Don’t know	Cross-sectional, 2017 and 2019	Effectiveness
Vendor	Store sales	Because of Vouchers 4 Veggies, my store makes more money	Yes No Don’t know	Cross-sectional, 2017 and 2019	Effectiveness
Vendor	Produce sales	Because of the Vouchers 4 Veggies, my store sells fruits and vegetables of better quality	Yes No Don’t know	Cross-sectional, 2019 only	Effectiveness
Vendor	Customer base	Because of Vouchers 4 Veggies, more customers shop at my store	Yes No Don’t know	Cross-sectional, 2017 and 2019	Effectiveness
Vendor	Customer base	Because of Vouchers 4 Veggies, new customers shop at my store	Yes No Don’t know	Cross-sectional, 2017 and 2019	Effectiveness
Vendor	Produce stocking	Are you displaying more fruit and vegetables as a result of Vouchers 4 Veggies?	Yes No	Cross-sectional, 2017 and 2019	Effectiveness
Vendor	Produce stocking	Are you stocking any new kinds of fruits or vegetables because of Vouchers 4 Veggies?	Yes No	Cross-sectional, 2017 and 2019	Effectiveness
Vendor	Ease of use	Vouchers 4 Veggies vouchers are easy to use at my store	Yes No Don’t know	Cross-sectional, 2017 only	Implementation
Vendor	Ease of use	Vouchers 4 Veggies staff are helpful	Yes No Don’t know	Cross-sectional, 2017 and 2019	Implementation
Vendor	Ease of use	Vouchers 4 Veggies staff quickly respond to my needs	Yes No Don’t know	Cross-sectional, 2017 and 2019	Implementation
Vendor	Ease of use	I receive quick reimbursements from Vouchers 4 Veggies for the vouchers I send in	Yes No Don’t know	Cross-sectional, 2017 and 2019	Implementation
Vendor	Ease of use	Overall, Vouchers 4 Veggies is easy to use	Yes No Don’t know	Cross-sectional, 2017 and 2019	Implementation
Vendor	Satisfaction	How likely would you recommend Vouchers 4 Veggies to a friend or colleague?	Scale of 0-10 (0, not likely—10, extremely likely)	Cross-sectional, 2017 and 2019	Implementation
Distribution partner	Ease of implementation	How easy or difficult is it to find participants at your site who are eligible for Vouchers 4 Veggies?	Very easy Easy Difficult Very difficult	Cross-sectional, 2016 and 2019	Implementation
Distribution partner	Ease of implementation	How easy or difficult is it to complete Vouchers 4 Veggies enrollment paperwork requirement?	Very easy Easy Difficult Very difficult	Cross-sectional, 2016 and 2019	Implementation
Distribution partner	Ease of implementation	How easy or difficult is it to continue distributing vouchers to participants after initial enrollment?	Very easy Easy Difficult Very difficult	Cross-sectional, 2019 only	Implementation
Distribution partner	Satisfaction	Please rate your level of satisfaction with the Vouchers 4 Veggies program	Very easy Easy Difficult Very difficult	Cross-sectional, 2019 only	Implementation
Distribution partner	Value	How helpful is Vouchers 4 Veggies as a resource for your clients?	Very helpful Helpful Somewhat helpful Not helpful	Cross-sectional, 2016 and 2019	Effectiveness

## Appendix 3 Maintenance survey methodology

In 2017, we surveyed a cross-sectional sample of former Vouchers 4 Veggies recipients using a mixed-methods approach to explore long-term impacts of the program on the primary outcomes of interest, including fruit and vegetable intake and food security status. We present the findings of the quantitative analysis in this manuscript. The eligibility criteria was as follows:

All individuals included in the evaluation were former participants who:

1. Were 6–12 months out of the program as of 1 July 2017 and had not received vouchers during that time
2. Completed a baseline and 6-month follow-up survey
3. Had re-enrolled in the program, but completed a survey prior to receiving vouchers again
4. Spoke and/or read English, Spanish, or Chinese

Given these criteria, 283 former participants were eligible to participate in this evaluation from 18 different distribution sites. Findings were based on surveys of 128 former participants. To maximize survey response rate, we administered surveys in person at voucher distribution sites, by phone, by mail, or a combination of the three. Surveys were available in three languages (English, Spanish, and Chinese) and consisted of a validated seven-item fruit and vegetable intake screener, the USDA’s six-item food security survey module, and closed- and open-ended questions addressing health status, healthy eating behaviors, and program satisfaction (see Appendix for full survey tool) (1, 2). We compiled and analyzed participant data at baseline, 6-month follow-up, and 6–12 months after program participation to assess change over time. All participants who completed a survey were compensated with a $10 gift card from a major pharmacy or grocery store.

Paired-sample *t*-tests were conducted to test the statistical significance of changes in fruit and vegetable intake and food security status between baseline, 6 months, and 6–12 months after program completion. All *P*-values are reported at the conventional significance level of <.05. All analyses were performed using Stata software (version 14.2; StataCorp, College Station, TX, United States), Microsoft Excel, and Microsoft Access.
